# USP15 regulates radiation-induced DNA damage and intestinal injury through K48-linked deubiquitination and stabilisation of ATM

**DOI:** 10.1186/s10020-024-00984-8

**Published:** 2024-11-09

**Authors:** Ruiqiu Zhu, Mingyue Li, Difan Wang, Chengzhi Liu, Liwei Xie, Yinyin Yang, Xuhao Gu, Kui Zhao, Ye Tian, Shang Cai

**Affiliations:** 1 Suzhou Key Laboratory for Radiation Oncology, Department of Radiotherapy and Oncology, The Second Affiliated Hospital of Soochow University, Suzhou, 215004 China; 2 Suzhou Medical College of Soochow University, Suzhou, 215000 China; 3 Department of Gastrointestinal Surgery, The Second Affiliated Hospital of Soochow University, Suzhou, 215004 China; 4 PRaG Therapy Center, Center for Cancer Diagnosis and Treatment, The Second Affiliated Hospital of Soochow University, Suzhou, 215004 China

**Keywords:** Radiation-induced intestinal injury, DNA damage response, Deubiquitination, USP15, ATM

## Abstract

**Background:**

Radiation-induced intestinal injury (RIII) interrupts the scheduled processes of abdominal and pelvic radiotherapy (RT) and compromises the quality of life of cancer survivors. However, the specific regulators and mechanisms underlying the effects of RIII remain unknown. The biological effects of RT are caused primarily by DNA damage, and ataxia telangiectasia mutated (ATM) is a core protein of the DNA damage response (DDR). However, whether ATM is regulated by deubiquitination signaling remains unclear.

**Methods:**

We established animal and cellular models of RIII. The effects of ubiquitin-specific protease 15 (USP15) on DNA damage and radion-induced intestinal injury were evaluated. Mass spectrometry analysis, truncation tests, and immunoprecipitation were used to identify USP15 as a binding partner of ATM and to investigate the ubiquitination of ATM. Finally, the relationship between the USP15/ATM axes was further determined via subsequent experiments.

**Results:**

In this study, we identified the deubiquitylating enzyme USP15 as a regulator of DNA damage and the pathological progression of RIII. Irradiation upregulates the expression of USP15, whereas pharmacological inhibition of USP15 exacerbates radiation-induced DNA damage and RIII both in vivo and in vitro. Mechanistically, USP15 interacts with, deubiquitinates, and stabilises ATM via K48-linked deubiquitination. Notably, ATM overexpression blocks the effect of USP15 genetic inhibition on DNA damage and RIII progression.

**Conclusions:**

These findings describe ATM as a novel deubiquitination target of USP15 upon radiation-induced DNA damage and intestinal injury, and provides experimental support for USP15/ATM axis as a potential target for developing strategies that mitigate RIII.

## Introduction

Radiotherapy (RT) was originally designed to kill cancer cells or slow their growth by inducing DNA damage and is a major therapeutic modality for treating abdominal or pelvic malignancies. However, the full therapeutic benefit of RT is limited to normal tissue injuries, particularly radiation-induced intestinal injury (RIII) (Ruysscher et al. 2019; Matta et al. 2019). Currently, our understanding of RIII mechanisms does not fully address the challenges posed by the significant dose-limiting toxicity of RT (Huh et al. 2020; Moraitis et al. 2023; Shakyawar et al. 2023). Therefore, exploring the novel mechanisms underlying RIII pathogenesis and developing effective intervention strategies are imperative.

One of the well-known defense mechanisms against radiation is the DNA damage response (DDR), which senses and repairs radiation-induced DNA damage, thus increasing the survival of intestinal epithelial cells (Chaves-Pérez et al. 2019). The DDR is tightly regulated by post-translational modifications (PTMs) of related proteins, including phosphorylation, acetylation, and ubiquitination (García-Giménez et al. 2021). For example, many aspects of the DDR are regulated by ubiquitination signaling, including the recruitment of effectors to DNA damage sites and the choice of the repair pathway (Clague et al. 2019; Das et al. 2020). Ubiquitination is highly dynamic and reversible. Deubiquitinases play critical roles in ubiquitin-directed signaling by catalytically removing ubiquitin from substrate proteins. Ubiquitin-specific peptidases (USPs) belong to a superfamily of deubiquitinases. In addition, several USPs such as USP4, USP5, USP7, and USP28 already participate in the DDR pathway, thereby regulating cellular sensitivity to radiation (Schwertman et al. 2016; Snyder, et al. 2021). Therefore, we suspect that ubiquitination and USPs may also play roles in RIII pathogenesis mechanisms.

DNA double-strand breaks (DSBs) are the most lethal form of radiation-induced DNA damage (R. X. Huang, et al. 2020; Pilié et al. 2019), and ataxia telangiectasia mutated (ATM) is a critical DDR enzyme that executes cellular responses to DSBs (Shi et al. 2021; Weitering et al. 2021). In response to DSBs, ATM is autophosphorylated and recruited to DNA lesion sites. Activated ATM phosphorylates a range of DDR-related proteins, including histone H2AX, the transcriptional regulator KAP1, and the effector kinase CHK2, thereby regulating the cell cycle DNA checkpoint response and promoting the repair of broken chromosomes (Lee, et al. 2021). Although how ATM regulates downstream targets of the DDR has already been extensively studied, how PTMs regulate the DDR in response to DSBs and whether USPs are integrated into ATM signaling remain unknown.

In this study, we employed transcriptomic sequencing to analyze an RIII model and found that the expression level of USP15 increased after radiation. In vitro and in vivo experiments subsequently revealed that USP15 regulates DNA damage and radiation-induced death in intestinal cells. Mass spectrometry revealed that USP15 directly interacts with ATM and stabilises it through K48-linked deubiquitination. We also found that the regulatory effect of USP15 on RIII was ATM dependent, as ATM overexpression blocked the impact of USP15 genetic inhibition on radiation-induced DNA damage and intestinal cell death. Taken together, our findings revealed a novel function of USP15 in modulating radiation-induced DNA damage and intestinal injury and revealed a crucial regulatory effect of USP15 on ATM. These findings suggest that the USP15/ATM regulatory axis is a potential novel target for protection against RIII.

## Materials and methods

### Antibodies and reagents

Antibodies and reagents were acquired from designated suppliers:

USP15 (66310, Cell Signaling Technology), USP15 (67557-1-lg, Proteintech), 8-OHdG (sc-66036, Santa cruz biotechnology), γ-H2AX (ab81299, Abcam), Ki67 (ab15590, Abcam), ATM (ab201022, Abcam), ATM (2715-1-AP, Proteintech), anti-mouse IgG (5415, Cell Signaling Technology), anti-rabbit IgG (3900, Cell Signaling Technology), and anti-GST (2624, Cell Signaling Technology), anti-Flag (66008, Proteintech), anti-Myc (16286, Proteintech), anti-Myc (2276, Cell Signaling Technology), anti-HA (3724, Cell Signaling Technology),

USP15-IN-1 (HY-148046, MedChemExpress), MG132 (S1748, Beyotime), cycloheximide (HY12320, MedChemExpress).

### Ethics statement and animals

Eight-week-old male C57BL/6J mice were purchased from Shanghai SLAC Laboratory Animal Co., Ltd. (Shanghai, China) and housed in a specific pathogen-free (SPF) animal facility at the soochow University Animal Centre. The mice were provided ad libitum access to water and standard chow and maintained on a 12-h light/dark cycle. All experimental procedures were conducted in compliance with the guidelines and protocols approved by the Animal Ethics Committee of Soochow University (SUDA202310A0542 ).

### Cell culture

The human intestinal epithelial cell line HIEC-6 was purchased from Meisen Chinese Tissue Culture Collections (Zhejiang, China). The human embryonic kidney (HEK293T) cells were obtained from Beyotime Company (Shanghai, China). The cells were cultured in Dulbecco’s modified Eagle medium (DMEM) supplemented with 10% fetal bovine serum (FBS) and 1% (v/v) penicillin-streptomycin at 37 °C under 5% CO2.

### Plasmids, lentiviral vectors, shRNA, and transfection

USP15, and ATM coding regions were tagged with Flag and Myc, respectively, and cloned into the pCDH-CMV-MCS-EF1α-Puro vector. Plasmids encoding HA-labeled ubiquitin and its mutants (#18712, #121151, #121152, #22902, #17607, #17606, #17605, and #17604) were acquired from Addgene. A point mutation in USP15 was performed using the QuikChange Mutagenesis Kit (Agilent Technologies). DNA sequencing was performed to verify these constructs. Lentiviral shRNA plasmids targeting USP15 were obtained from Shanghai Genechem (Shanghai, China). The sequences were as follows: shRNA#1, 5’-GATACAGAGCACGTGATTATT-3’, shRNA#2, 5’- ATTTGTGGATGTTGGTCATTT − 3’. Transfection of the plasmids and shRNA was performed using Lipo3000 (Invitrogen).

### Irradiation protocol

The mice were anesthetized with an intraperitoneal injection of pentobarbital sodium (approximately 40 mg/kg) and placed on a platform to receive 14 Gy of whole abdominal irradiation (WAI) using an X-RAD 320iX Biological Irradiator (Precision X-ray, North Branford, CT, USA) at a dose rate of 1.0 Gy/min. A 3-cm abdominal area from the xiphoid process of the sternum to the symphysis pubis covering the gastrointestinal tract was irradiated, and other parts of the body were shielded by a 5 cm-thick lead block. The exact dosimetry values can be found in Bell et al(Bell et al. 2022).

Vitro model of intestinal epithelial cell injury was established by irradiating HIEC cells at the dose rate of 1.1 Gy/min using an X-RAD 320iX Biological Irradiator (Precision X-ray, North Branford, CT, USA).

### RNA-Seq assay

Samples were isolated, gently washed with DEPC-treated water (10601ES76, YEASEN), frozen and then used for transcriptome RNA-Seq. Total RNA extraction, RNA integrity evaluation, library construction, and sequencing were performed according to the manufacturer’s standard protocol. RNA-seq and analysis were conducted by OE Biotech Co., Ltd. (Shanghai, China). Diferentially expressed genes (DEGs) were identifed usingthe absolute value of log2 (ratio) ≥ 1 as the threshold. Te t test threshold (P values < 0.05) and fold-change threshold (> 1.5 or < 0.5) were set as the thresholds for signifcantly DEGs.

### Histological staining

Ileum tissues were collected 6 h and 3 days after radiation. Tissue samples were fixed in 4% paraformaldehyde, embedded in paraffin, cut into 4-µm sections, and stained with hematoxylin-eosin (H&E) and periodic acid-Schiff (PAS). For PAS staining, 4-µm sections were deparaffinized, hydrated, and placed in a periodic acid solution (1%) for 5 min at room temperature. After a wash, the slide was immersed in Schiff’s reagent for 5 min at 60 °C and washed in hot water for 5 min. H&E-stained sections were viewed under an optical microscope, and villus height was analyzed using ImageJ software (Version 1.8.0.112, National Institutes of Health, Bethesda, MD, USA).

### Colony formation assay

The HIEC cells were seeded in triplicate in 6-well plates at the density of 100–2000 cells/well depending on the radiation dose. After overnight culture, the cells were pretreated with 0 or 1 µM USP15-IN-1 for 30 min, followed by 0, 2, 4, 6, and 8 Gy X-ray radiation. The cells were cultured for 1–2 weeks and stained with crystal violet, and the viable colonies containing at least 50 cells were counted.

### Immunofluorescent (IF) staining

Cells were seeded in confocal dishes at the appropriate density. After fixation with 4% paraformaldehyde, permeabilization with 0.5% Triton X-100, and blocking with regular goat serum, the cells were incubated with the indicated primary antibodies at 4 °C overnight. The cells were washed three times with PBS and then incubated with Alexa Flour 488- or 594-labeled secondary antibodies (A11001, A11008, A11037, and A11032; Thermo Fisher). Images were then captured with a confocal microscope and analyzed using LAS X software.

### Mass spectrometry analysis of USP15 interacting proteins

HIEC-6 cells transfected with Flag-USP15 were subjected to immunoprecipitation (IP) with an anti-Myc antibody or IgG control. Immune complexes were then incubated with Protein A/G Agarose (P2197, Beyotime). After washing 4 times, the immunoprecipitates were boiled in 1X loading buffer and resolved using SDS-PAGE. MS analysis was then performed by Guangzhou Saicheng Biotechnology company (Guangzhou, China).

### Duo-link proximity ligation assay (PLA)

For the PLA assay, 5 × 10^3^ cells were seeded into a confocal dish. Following fixation with 4% paraformaldehyde, the cells were permeabilized, blocked and probed with the indicated antibodies. Afterwards, the Duolink^®^ In Situ Red Mouse/Rabbit kit (DUO92101, Sigma Aldrich) was used to detect the PLA foci according to the manufacturer’s protocol.

### Immunoprecipitation (IP)

For IP analysis, cell lysis buffer (P0013, Beyotime) containing PMSF and phosphatase inhibitor cocktail was used to lyse the cells for IP analysis (P1081, Beyotime). Cell lysates were first precleared with protein A/G agarose, and then were incubated with the indicated primary antibodies at 4 °C overnight. The next day, 40 µl of protein A/G-agarose beads were added and incubated for 4 h at 4 °C. After washing three times, the mixture was resuspended. After boiling and centrifugation to pellet the agarose beads, supernatants were subjected to IB analysis.

### Co-immunoprecipitation assay

Lysis buffer (Beyotime, catalog P0013), which contained a protease inhibitor cocktail (MCE, catalog HY-K0010), was used for lysis. The indicated antibodies were used in cell lysates for immunoprecipitation at 4 °C overnight and then incubated with protein A/G (Beyotime, catalog P2055) at 4 °C for 3 h. Then the cell lysates were washed with lysis buffer four times and then analyzed by IB.

### Protein purification and GST pull-down

Bacterially expressed GST, GST-ATM was incubated with Flag-USP15WT or Flag-USP15^C298A^ lysed from HEK293T cells using GST Protein Interaction Pull-Down Kit (Thermo Scientific, catalog 21516). The complex was then analyzed by Coomassie Brilliant Blue.

### In vivo and in vitro ubiquitination assay

Denaturing immunoprecipitation (d-IP) was used to carry out an in vivo ATM ubiquitination experiment. After treatment with 20 µM MG132 for 6 h, cells were collected using denatured lysis solution (1.5% β-mercaptoethanol, 62.5 mM Tris-HCl pH 6.8, 10% glycerol, 2% SDS) and boiled for 10 min. After centrifugation for 5 min, immunoprecipitation (IP) and immunoblotting (IB) were performed with indicated the antibodies.

For the in vitro ubiquitin assay of ATM, purified ATM was incubated with Flag-USP15, UBE1 (SRP0400, Sigma-Aldrich), UBE2D3 (ab269098, Acbam) and the indicated His-tagged-ubiquitin mutants at 37 °C for 45 min in 40 µl reaction buffer (25 mM Tris pH 7.4, 5 mM MgCl2 and 2 mM DTT). The supernatant was then collected by centrifugation for 5 min and then boiled at 95 °C for 5 min after the addition of SDS loading buffer. Myc beads were used to pull down ubiquitinated ATM. The eluted proteins were analyzed by IB.

### Statistical analysis

Analyses were carried out using GraphPad Prism software (Version 7.0). Data are represented as the mean ± SD. Two-tailed Student’s t-tests and one-way ANOVAs were utilized in the data analysis for the article. A Kaplan-Meier model was used to conduct survival analysis. A P-value less than 0.05 was considered to be statistically significant.

## Result

### USP15 is upregulated in both the mouse and cell models of RIII

**Fig. 1:**
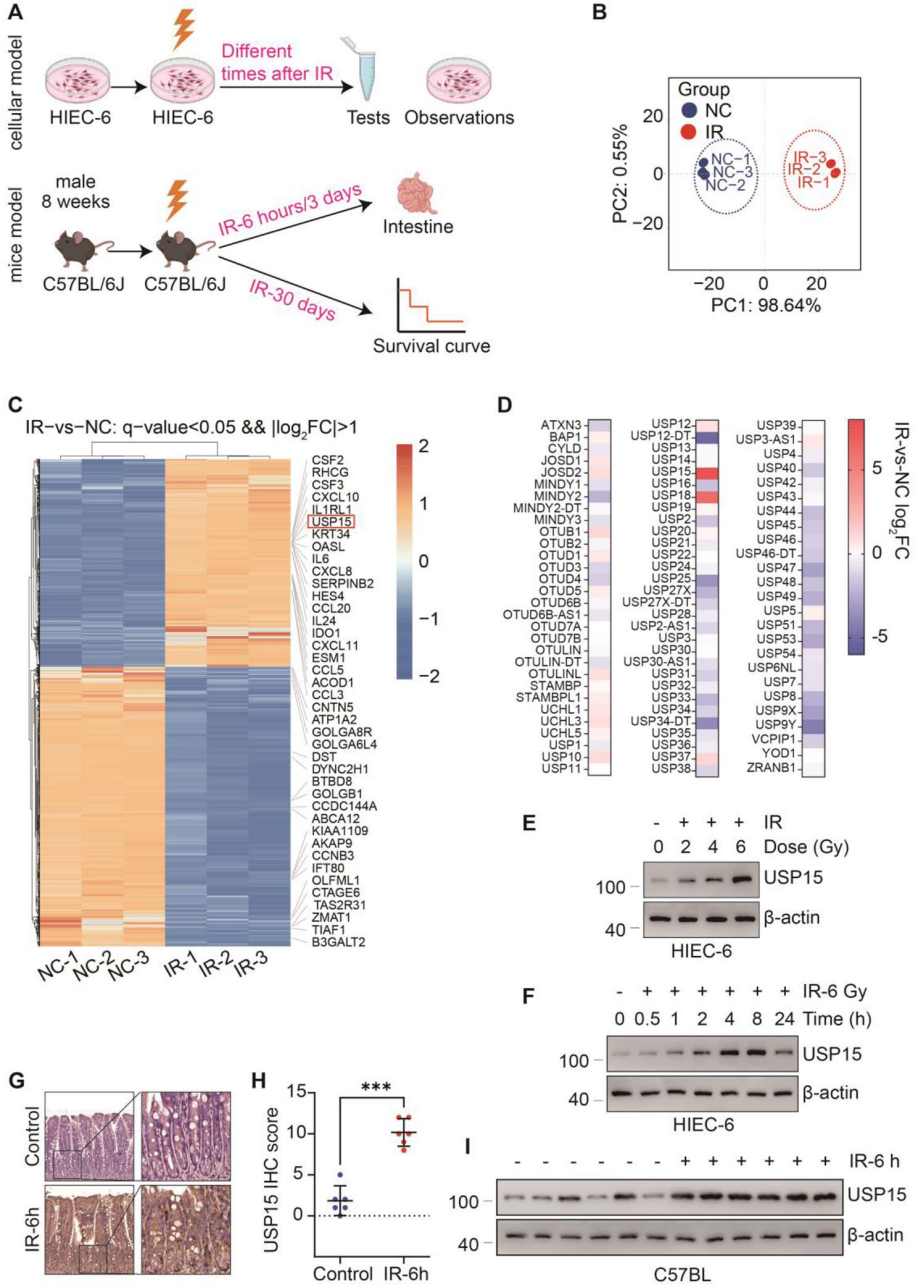
USP15 is upregulated in RIII model. **a** Schematic representation of in vivo and in vitro models of RIII. **b** Principal component analysis (PCA) score plot from NC and IR-6 h after ionization radiation. **c** Hierarchical clustering heatmap of genes of NC and IR-6 h after ionization radiation. Red represents increased expression, while blue represents decreased expression. **d** Heatmap showing relative gene expression levels of the DUB family. **e** Proteins of HIEC-6 cells treated with 0, 2, 4, 6 Gy irradiation were extracted and Western blot was performed to detect the protein level of USP15. β-actin was used as a loading control. **f** Protein expression of USP15 in irradiated HIEC-6 cells at different time points. **g**,** h** Representative IHC images of USP15 expression in Control and IR-6 h after 14 Gy WAI mice tissues. Scale bars: 20 μm. Data are represented as the mean ± SD. Statistical analysis was performed using Student’s t-test and two-sided t-test (H), *p < 0.05; ***p < 0.001; ****p < 0.0001. **i** Protein levels of USP15 between NC- and IR-6 h in mice intestine

We previously established an RIII mouse model via using whole-abdomen irradiation (WAI) (Fig. 1a). To explore the potential role of ubiquitination in RIII progression, samples from the control and ionization radiation groups were collected for RNA sequencing (RNA-seq). The transcriptomes of these two groups were significantly different (Fig. 1b). The RNA-seq data indicated that USP15 was significantly upregulated after irradiation (Fig. 1c). Furthermore, we observed that USP15 was the most highly expressed USP in response to radiation (Fig. 1d). Consistently, the Western Blot results revealed remarkable upregulation of USP15 protein levels in human intestinal epithelial cells (HIECs) after radiation in a time- and dose-dependent manner (Fig. 1e, f). We also detected much greater USP15 protein expression in the mouse intestine after irradiation via Western B lotting and immunohistochemistry (Fig. 1g–i). These results indicate that the intestinal expression of USP15 can be induced by radiation, suggesting that USP15 may play a regulatory role in the pathological progression of RIII.

### Pharmacologic inhibition of USP15 exacerbates radiation-induced cell death and DNA damage in vitro

**Fig. 2:**
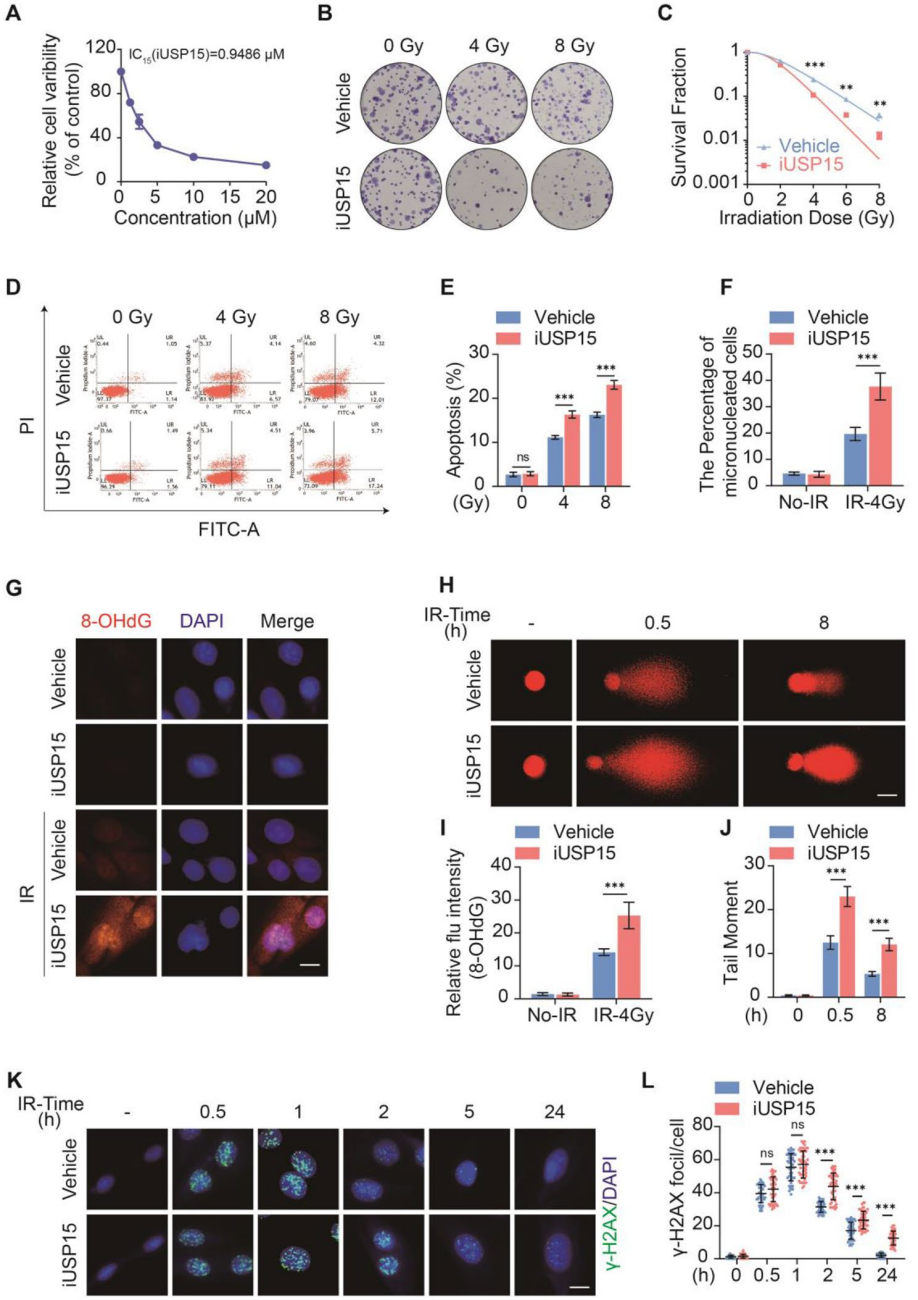
Pharmacologic inhibition of USP15 exacerbates RIII in cell model. **a** Cell viability of HIEC-6 cells after treated with USP15-IN-1 for 24 h. **b**, **c** Clonogenic survival assay of HIEC-6 cells pretreated with DMSO or 0.1 µM USP15-IN-1 following 2, 4, 6, or 8 Gy radiation. **d** Cell apoptosis was measured using Annexin V staining in HIEC-6 cells treated with 0.1 µM USP15-IN-1 or vehicle 0.5 h before 4 or 8 Gy irradiation. **e** Percentage of apoptotic cells in each group. **f** USP15-IN-1 increased micronucleus formation in HIEC-6 cells upon 2 Gy X-irradiation. **g**, **i** Representative IF images showing the expression of 8-OHdG in HIEC-6 cells pretreated with DMSO or 0.1 µM USP15-IN-1 0.5 h before 4 Gy IR. The fluorescence intensity of 8-OHdG in HIEC-6 cells was quantified. Scale bar: 10 μm. **h**, **j** Comet assay on HIEC-6-Vehicle/iUSP15 cells at different times after 2 Gy X-irradiation. The tail moments were quantified using CometScore. At least 500 cells were scored per sample. Scale bars: 10 μm. **k**, **l** Representative immunofluorescence images of γ-H2AX in HIEC-6-Vehicle/iUSP15 cells after 2 Gy X-irradiation and quantification of the number of γ-H2AX foci per cell. At least 300 nuclei were scored. Scale bars: 10 μm. Data are presented as mean ± SD. n=3-5. **P < 0.01, ***P < 0.001

To clarify the role of USP15 in RIII pathological progression, a specific USP15 inhibitor USP15-IN-1 (iUSP15) was used, and a low concentration of iUSP15 (IC_15_, 1 µM) was selected for further study based on the CCK-8 assay results (Fig. 2a). We found that iUSP15 increased both radiation-induced clonogenic cell death (Fig. 2b, c) and apoptosis (Fig. 2d, e) in HIECs via colony formation assays and flow cytometry, respectively. We subsequently detected important DNA damage indicators, such as micronuclei formation, comet assay, γH2AX foci (indicating DSBs), and 8-OHdG staining (indicating DNA oxidative damage). As expected, iUSP15 increased all these DNA damage indicators in HIECs after irradiation (Fig. 2f–l). These data suggest that pharmacological inhibition of USP15 exacerbates DNA damage cell death, in HIECs after radiation.

### Pharmacologic inhibition of USP15 exacerbates RIII and DNA damage in vivo

**Fig. 3:**
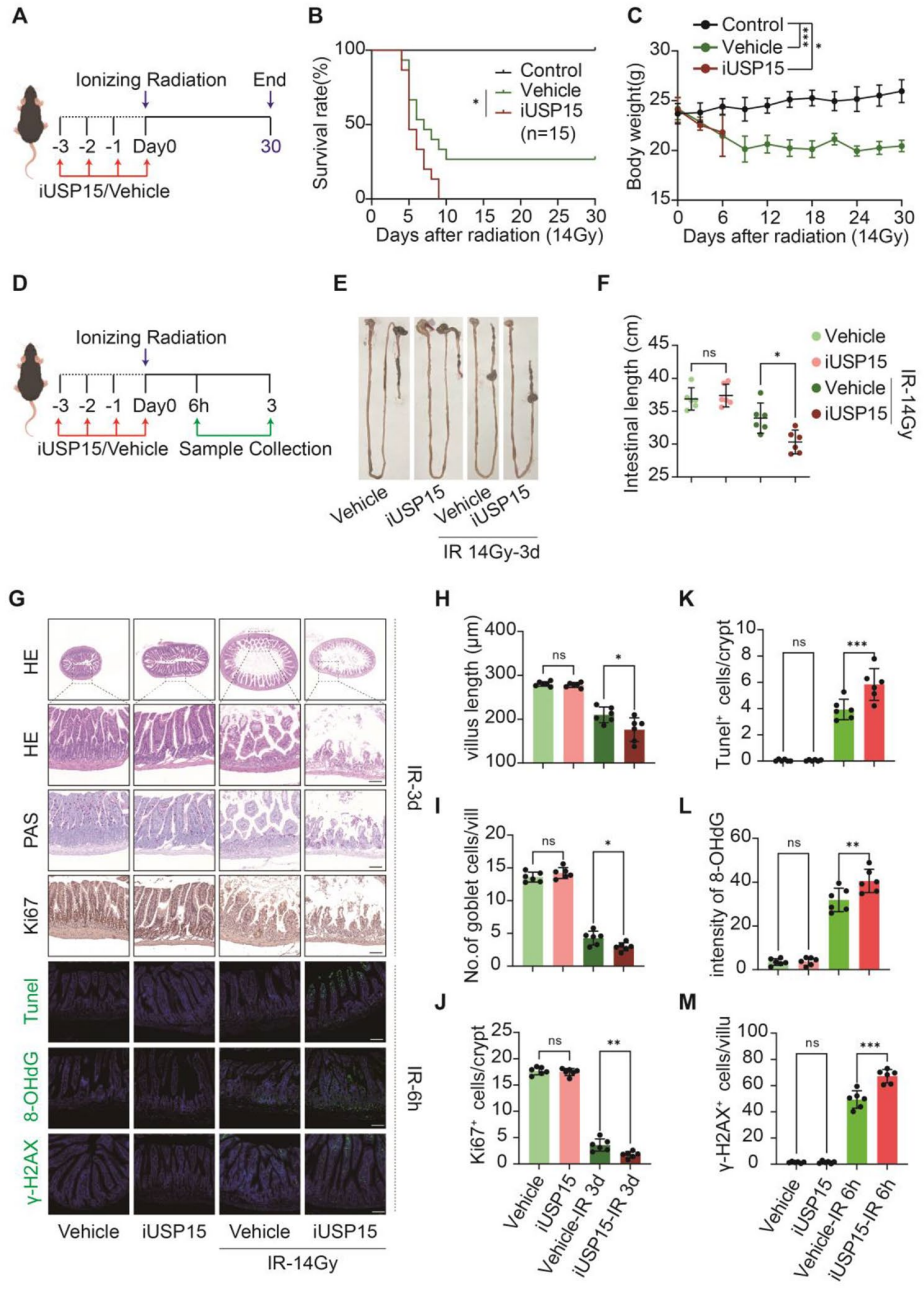
Pharmacologic inhibition of USP15 exacerbates RIII in mice model. **a** C57 BL/6J mice received an intraperitoneal injection of 0.1 mg/kg USP15-IN-1 for 3 consecutive days before 14 Gy WBI and 30 min after WBI. Similarly, the control mice were exposed to 14 Gy WBI and received injections of vehicle (an equivalent volume of normal saline) at the same frequency. **b** Kaplan-Meier survival analysis of mice after 14 Gy WBI (*n* = 15 per group). ***P < 0.001 for vehicle vs. 0.1 mg/kg USP15-IN-1. **c** Body weights of irradiated mice in each group after 14 Gy WBI. **d** Small intestinal tissue samples were collected from the control and irradiated mice treated with vehicle/USP15-IN-1 at 6 h and 3.5 days after 14 Gy WBI. **e**, **f** Intestine length of mice in each group at 3 days post-WAI. **g** Representative H&E-stained, PAS-stained, Ki-67-stained, TUNEL-stained, 8-OHdG-stained and γ-H2AX-stained intestinal sections of various groups. Scale bar: 100 μm. **h** Villus height of mice at 3 days post-WAI. **i** Number of PAS^+^ cells per villus at 3 days post-WAI. **j** Number of Ki67^+^ cells per crypt and surviving crypts per circumference at 3 days post-WAI. **k** Number of TUNEL^+^ cells per crypt at 6 h post-WAI. **l** intensityof 8-OHdG at 6 h post-WAI. **m** Number of γ-H2AX^+^ cells per villus at 6 h post-WAI. *P < 0.05; **P < 0.01; ***P < 0.001; *n* = 6/group

We further investigated the effect of USP15 on RIII progression in vivo. The mice were intraperitoneally administered either iUSP15 (“iUSP15” group) or saline (“Control” and “Vehicle” groups) before WAI (Fig. 3a). First, we evaluated the effects of iUSP15 on survival and reported that 26.7% (4/15) of the Vehicle group mice survived WAI-induced death. In contrast, none of the iUSP15 group mice survived beyond nine days after WAI (Fig. 3b). Next, the effect of iUSP15 on the intestinal structure was evaluated. We observed a shortened intestinal length, reduced villus length, and decreased numbers of goblet cells and Ki67-positive cells. In addition, the number of apoptotic cells in the mouse intestine increased after WAI (Fig. 3d–k). As expected, radiation-induced damage to the intestinal structure was exacerbated by iUSP15 (Fig. 3d–k). Consistent with the in vitro findings described above, iUSP15 also increased the number of 8-OHdG-stained and γH2AX foci in the mouse intestine after radiation (Fig. 3g, l, and m). These data suggest that the pharmacological inhibition of USP15 exacerbates RIII and DNA damage in vivo after radiation.

### USP15 interacts with ATM via the D3 domain

**Fig. 4:**
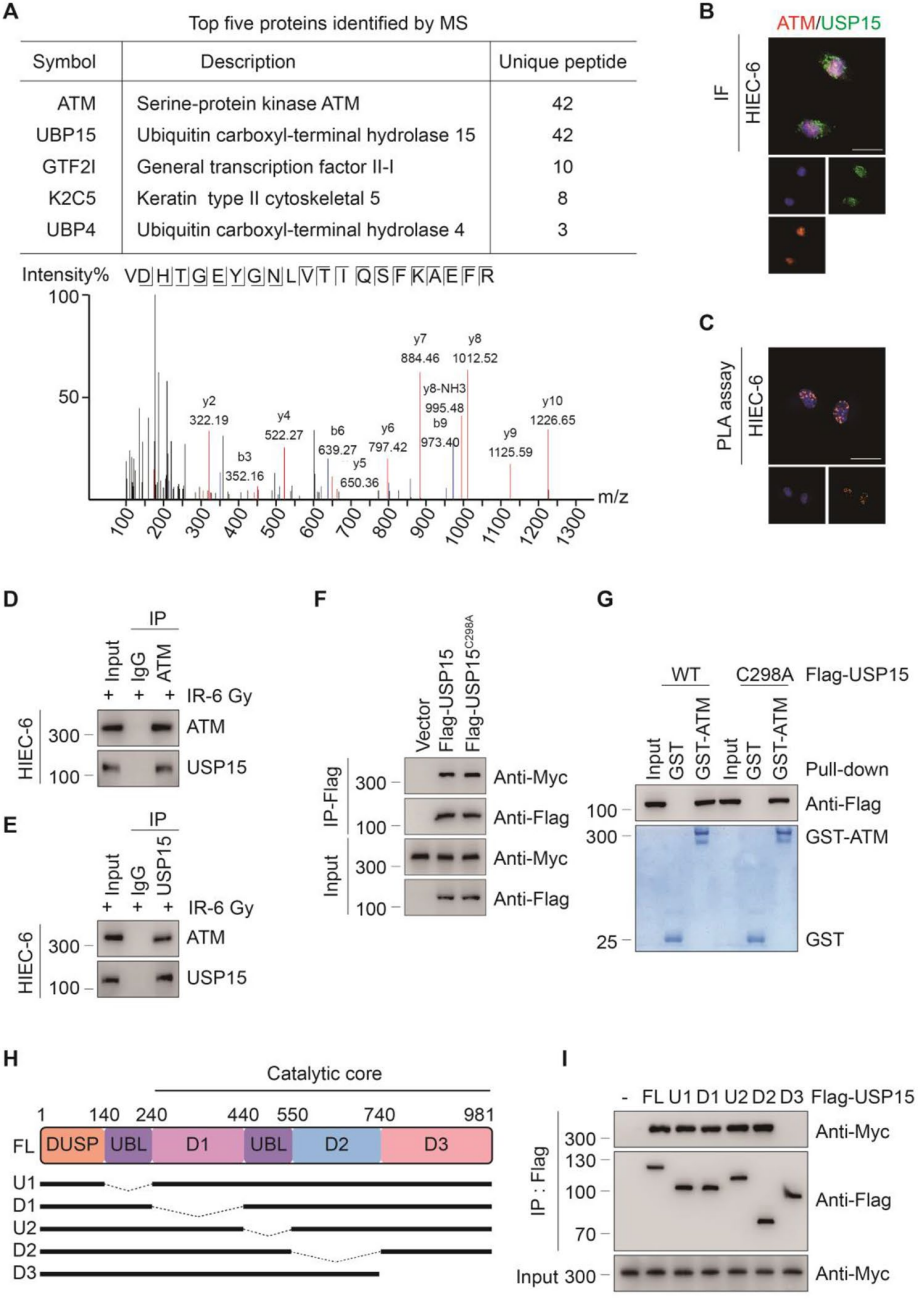
USP15 directly interacts with ATM. **a** Peptides of USP15 identified by mass spectrometry (MS) analysis. **b** Confocal assay showing co-localization of Flag-USP15 (green) and ATM (red)in HIEC-6 cells. Nuclei were counterstained with DAPI (blue). Scale bar: 20 μm. **c** Images of PLA for endogenous ATM and USP15 in HIEC-6 cells transfected as indicated. Scale bar: 20 μm. **d**, **e** Co-IP validation of the interaction between USP15 and ATM in HIEC-6 cells. **f** Co-IP showing ATM interaction with USP15 WT and C298A mutant. Anti-Flag antibody was used to bind Flag-tagged USP15 WT or C298A. Anti-Myc antibody was used to bind Myc-tagged ATM. **g** The GST-Pulldown assay showing the direct interaction between USP15 and ATM. **h** Schematic structures of USP15, together with their truncated mutants. **i** Co-IP showing that the C terminal of USP15 is essential for the interaction with ATM

To further clarify the mechanism by which USP15 regulates radiation-induced intestinal DNA damage and injury, mass spectrometry was used to identify proteins to which USP15 potentially binds. The most prominent was protein kinase ATM, which belongs to the phosphoinositide 3-kinase (PI3K) family of proteins and acts as a key upstream mediator of the DDR (Fig. 4a). We subsequently confirmed that USP15 could bind to ATM both endogenously and exogenously via confocal microscopy, dual-link proximity ligation assay (PLA), and reciprocal coimmunoprecipitation (Co-IP) experiments (Fig. 4b–e). Because USP15 is a deubiquitylating enzyme, we investigated whether its deubiquitylating enzyme activity was required for its interaction with ATM. We found that either the Flag-USP15 wild-type (WT) or the Flag-USP15 C298A mutant (a catalytically inactive mutant of USP15) were able to bind Myc-ATM (Fig. 4f) or GST-ATM (Fig. 4g) in HEK293T cells. Therefore, the direct binding between USP15 and ATM is deubiquitination independent. USP15 contains a DUSP domain at its amino terminus, two UBL domains in the middle, and a carboxyl terminus. Next, we tested which region of USP15 is responsible for its interaction with ATM by expressing ATM together with USP15 or its truncated mutants (Fig. 4h) and found that the USP15 D3 deletion mutant (deletion residues 740–981) abolished the binding of USP15 with ATM (Fig. 4i). Taken together, these results suggest that USP15 can directly interact with ATM via its D3 domain, but independent of its deubiquitination activity.

### USP15 stabilises ATM via K48-linked deubiquitination

**Fig. 5:**
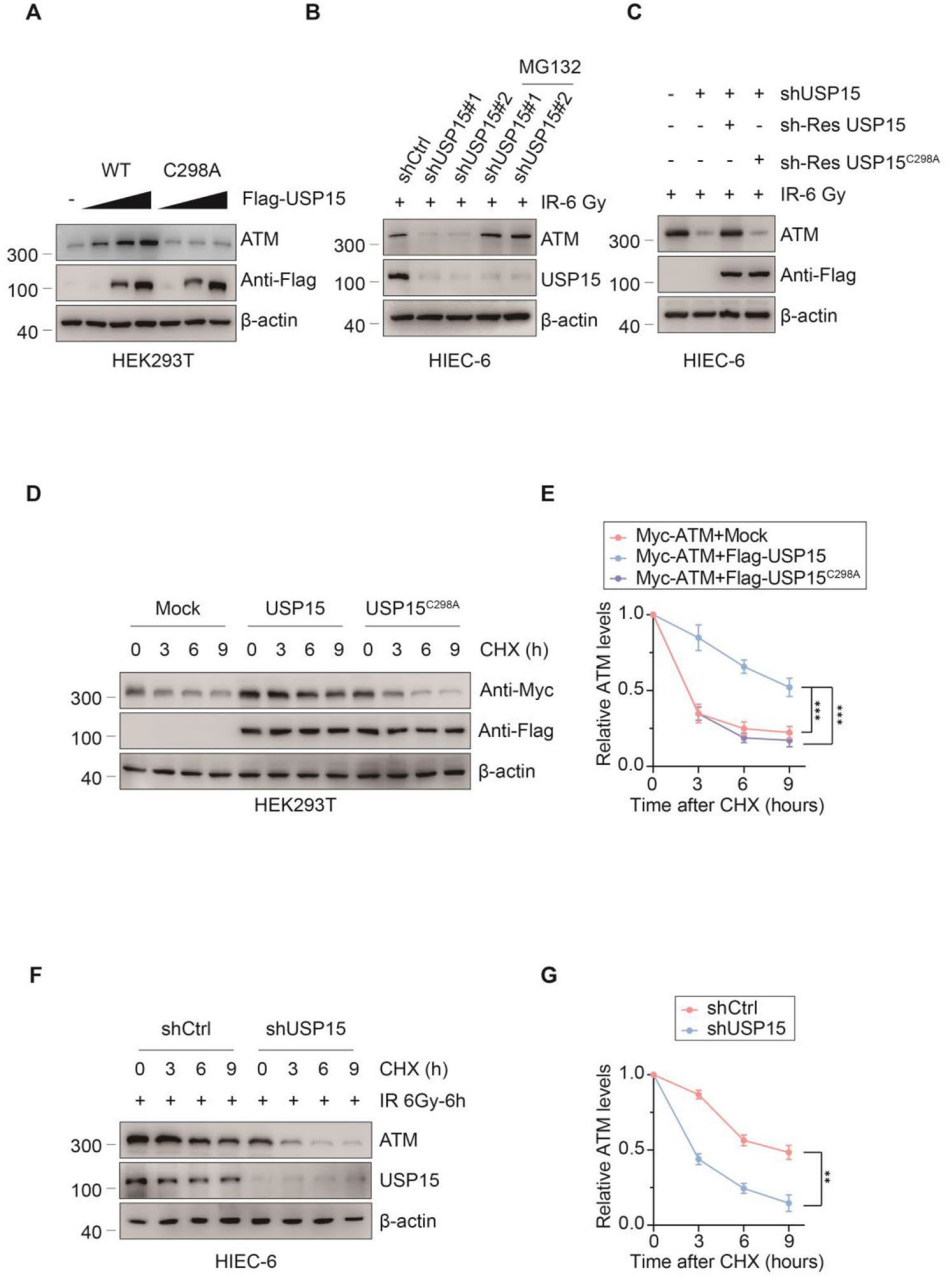
USP15 maintains ATM stability. **a** Western blotting showing that transiently transfecting of USP15-overexpressing plasmid altered the expression of ATM in a dose-dependent manner. **b** Western blotting shows that knockdown of USP15 attenuated the expression of ATM in HIEC-6 cells, whereas treatment with MG-132 (20 µM) abolished the effect of the knockdown of USP15 in HIEC-6 cells thus increasing the expression of ATM. **c** Western blotting showing that the overexpression of an shRNA-resistant WT, but not C298A mutant, USP15 altered the effect of the knockdown of USP15 in HIEC-6 cells thus increasing the expression of ATM. **d**,** e** Western blotting showing that the overexpression of wild-type USP15 (USP15 WT) but not the catalytically inactive USP15 mutant (USP15 C298A) stabilized ATM. ***P < 0.001. **f**,** g** Western blotting showing that the knockdown of USP15 in HIEC-6 cells resulted in accelerated degradation of ATM.*** *P* < 0.001

Since USP15 can interact with ATM, we next hypothesized that ATM is a prime target of USP15 and investigated whether USP15 plays a role in ATM stability. We found that the forced expression of Flag-USP15 WT, but not the catalytically inactive USP15 mutant (Flag-USP15 C298A mutant), upregulated ATM protein levels in a dose-dependent manner (Fig. 5a). In contrast, USP15-knockdown cells presented decreased ATM protein levels. However, this effect was blocked by the proteasome inhibitor MG132 (Fig. 5b). We also found that USP15 knockdown-mediated ATM protein reduction was almost completely restored upon overexpression of shRNA-resistant Flag-USP15 WT, but not upon overexpression of the catalytically inactive mutant USP15 (Fig. 5c). Finally, a cycloheximide (CHX) chase assay was performed. We observed that the overexpression of USP15 WT, but not its catalytically inactive mutant USP15 C298A, prolonged the half-life of the ATM protein (Fig. 5d, e). In contrast, knockdown of UPS15 markedly shortened the half-life of the ATM protein (Fig. 5f, g). Taken together, these results indicate that USP15 regulates ATM stability by preventing its proteasomal degradation and that this effect requires the catalytic activity of USP15.

**Fig. 6:**
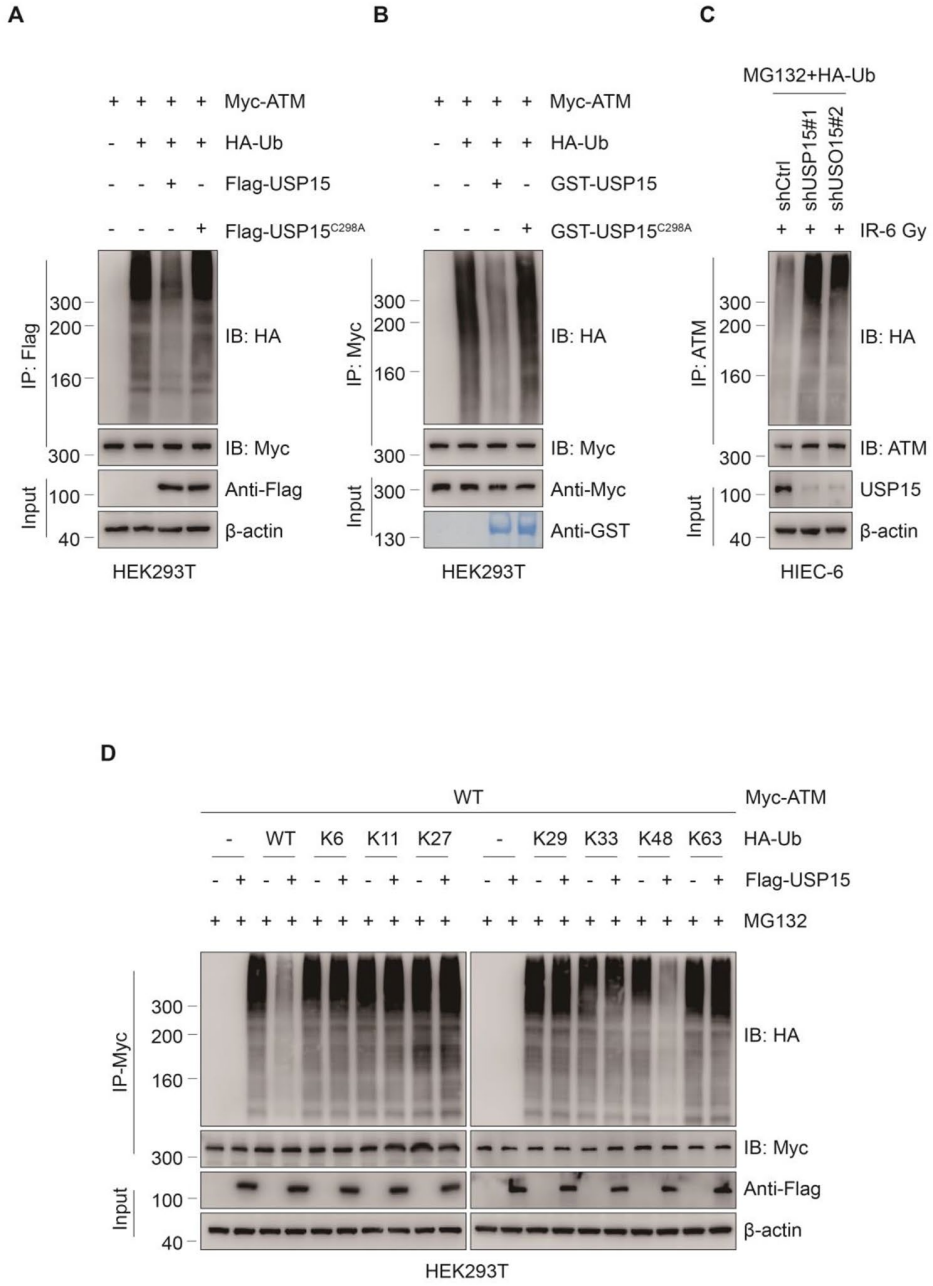
USP15 deubiquitinates ATM. **a** Co-IP showing that USP15 WT, but not C298A, reduced polyubiquitinated ATM. Anti-HA antibody was used to bind HA-tagged Ub to indicate ubiquitination. **b** GST Pulldown assay showing that ATM is a direct deubiquitinated substrate of USP15. **c** Co-IP showing that the knockdown of USP15 increased accumulation of polyubiquitinated ATM in HIEC-6 cells. Anti-HA antibody was used to bind HA-tagged Ub to indicate ubiquitination. **d** Co-IP showing that USP15 efficiently disassembled K48- linked polyubiquitylation of ATM but had no significant effect on monoubiquitylation or the K6, K11, K27, K29, K33, K63-linked polyubiquitylation of ATM. Anti-HA antibody was used to bind HA-tagged Ub to indicate ubiquitination

Since protein stability is controlled mainly by the ubiquitin proteasome system (UPS), we investigated whether USP15 plays a role in ATM ubiquitination. To test this hypothesis, we overexpressed Myc-ATM and HA-Ub via Flag-USP15 WT or Flag-USP15 C298A in HEK293T cells and conducted denaturing-IP (d-IP) assays. The results revealed that cells overexpressing USP15 WT, but not USP15 C298A, presented lower levels of ATM polyubiquitination (Fig. 6a). An in vitro ubiquitination assay further demonstrated that USP15 targets ATM for ubiquitination (Fig. 6b). In contrast, HIECs in which USP15 was knocked down via two independent shRNAs presented increased ATM ubiquitylation (Fig. 6c). Since polyubiquitination can occur through seven different lysine (Lys) residues on ubiquitin (K6, K11, K27, K29, K33, K48, and K63), we used a panel of ubiquitin mutants in which only one Lys residue was retained. We found that USP15 decreased only the K48-linked polyubiquitination of ATM. These results suggest that USP15 stabilises ATM by mediating the direct K48-linked ubiquitination of ATM.

### The regulation effect of USP15 on radiation-induced intestinal cell death and DNA damage depends on ATM

**Fig. 7:**
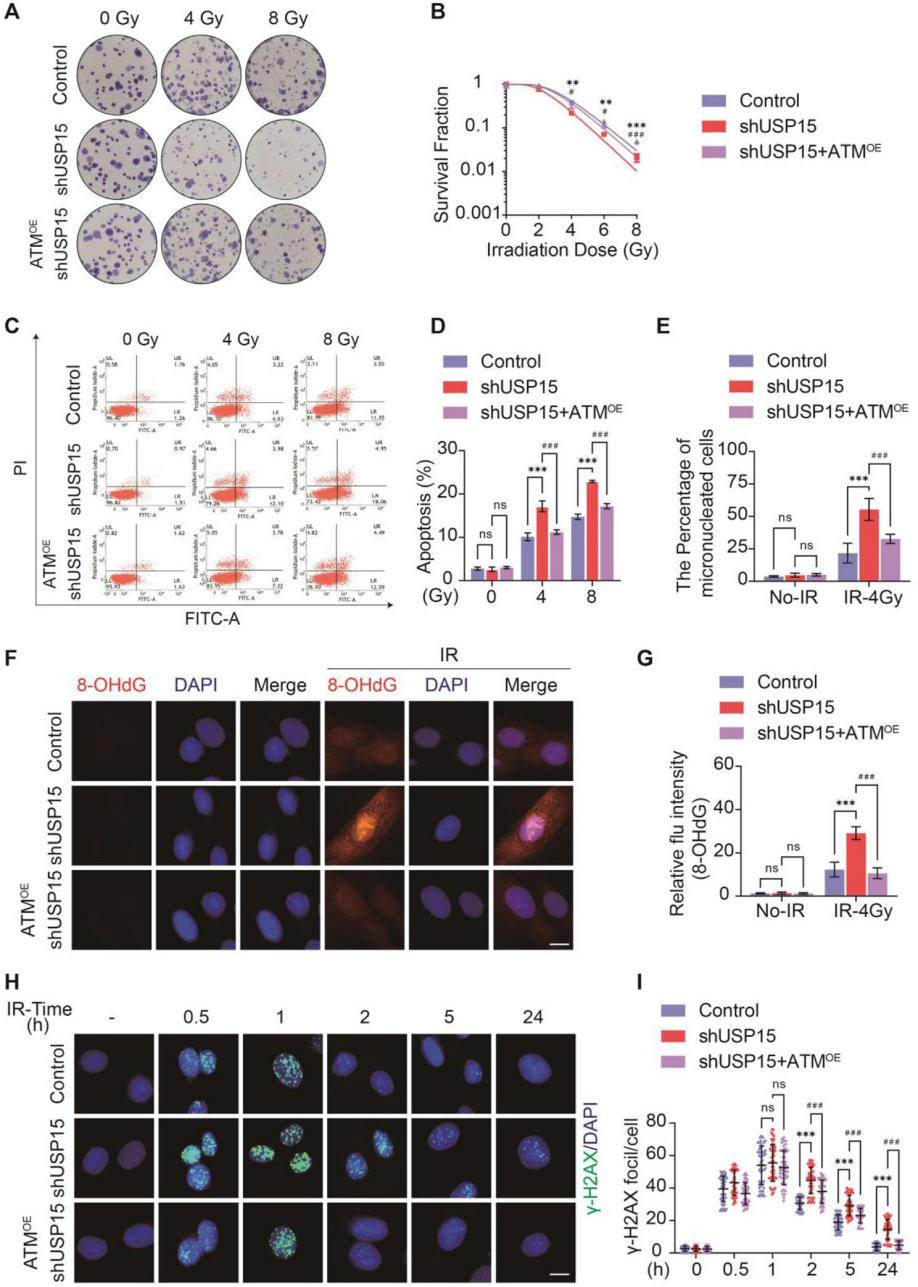
USP15 regulates RIII via ATM. **a**,** b** Clonogenic survival assay of HIEC-6 cells following 2, 4, 6, or 8 Gy radiation. **c**,** d** Cell apoptosis was measured using Annexin V staining in HIEC-6 cells following 4–8 Gy irradiation. **e** micronucleus formation in HIEC-6 cells upon 2 Gy X-irradiation. **f**,** g** Representative IF images showing the expression of 8-OHdG in HIEC-6 cells following 4 Gy IR. The fluorescence intensity of 8-OHdG in HIEC-6 cells was quantified. Scale bar: 10 μm. **h**,** i** Representative immunofluorescence images of γ-H2AX in HIEC-6-Control/shUSP15/shUSP15 + ATM^OE^ cells after 2 Gy X-irradiation and quantification of the number of γ-H2AX foci per cell. At least 300 nuclei were scored. Scale bars: 10 μm.Data are presented as mean ± SD. n=3-5. **P < 0.01, ***P < 0.001, Control group vs. shUSP15 group. ^##^*P* < 0.01, ^###^*P* < 0.001, shUSP15 group vs. shUSP15 + ATM^OE^ group

Considering the regulatory effects of USP15 on ATM deubiquitination and stability, we aimed to determine the role of the USP15/ATM axis in RIII progression. Consistent with the impact of pharmacological USP15inhibition on RIII, genetic inhibition of USP15 also exacerbated RIII. This is reflected by increased reproductive cell death, apoptosis, and DNA damage in HIECs after irradiation (Fig. 7a–j). However, ATM overexpression nearly completely blocked these effects (Fig. 7a–j). In summary, these data suggest that USP15 regulates radiation-induced DNA damage and intestinal injury in an ATM-dependent manner.

## Discussion

**Fig. 8:**
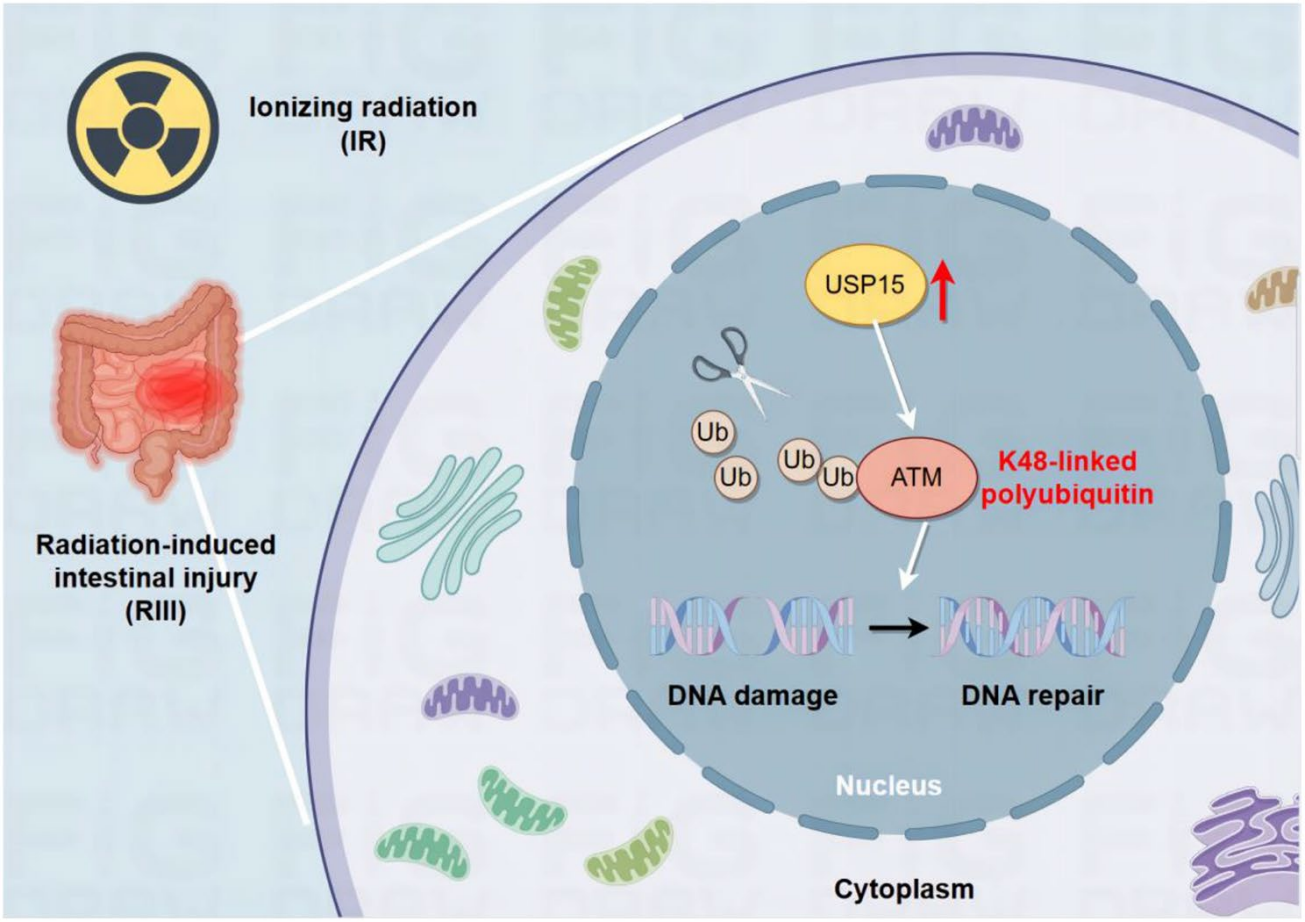
Working model for the regulatory effects of USP15 on the stabilization of ATM in RIII. The overexpression of USP15 induced by IR increases the level of ATM K48-linked deubiquitination in RIII models. This upregulates the stability of ATM proteins to influence the DNA damage response. Ultimately, the USP15/ATM regulatory axis was shown to modulate radiation-induced DNA damage and intestinal damage

In this study, we identified USP15 as a key regulator of proteasomal degradation of the DDR and ATM in intestinal cells. Our data revealed that USP15 directly interacts with ATM and induces its K48-linked deubiquitination, thereby protecting it from proteasomal degradation. Moreover, we demonstrated that ATM is essential for USP15 -mediated regulation of radiation-induced DNA damage and intestinal injury. These data highlight the pivotal role of the USP15/ATM axis in RIII progression (Fig. 8), suggesting a novel potential strategy for effective intervention.

Approximately half of cancer patients will receive radiotherapy during their whole course of disease, and 50% are irradiated in the abdominal or pelvic cavity. Despite progress in the development of more advanced radiotherapy techniques and equipment, up to 80% of those patients who receive abdominal or pelvic radiotherapy will develop RIII due to inevitable irradiation of the intestine. Unfortunately, the intestine remains one of the most radiosensitive organs in the body, as it has rapidly proliferating and differentiating cells. However, although various of potential countermeasures, including radioprotectors and mitigators, are under development but are still not approved for clinical application. Therefore, the prevention and management of RIII has become a major and urgent issue for both radiation oncologists and radiation biologists.

DNA damage is widely recognized as the primary biological effect induced by radiation, which triggers DDR signaling. Various DNA damage repair signaling pathways are subsequently activated, including base excision repair, nucleotide excision repair, mismatch repair, homologous recombination repair (HRR) and nonhomologous end-joining (NHEJ) (R. Huang, et al. 2021). In contrast, failure of DNA damage repair causes the death of damaged cells and ultimately leads to normal tissue injuries, including RIII (Zhao et al. 2022). Proteins are the fundamental units of cellular activity, and ubiquitin widely modifies proteins, thereby regulating most cellular functions. Accumulating evidence indicates that ubiquitin enzymes regulate DNA damage repair after radiation exposure, mainly by regulating the ubiquitination and stability of key regulators of the DDR (Cao et al. 2024; Koo et al. 2023; Zhu et al. 2021). One example is DSB-induced K48-linked ubiquitylation of the Ku complex, which causes its elimination from DNA, thus regulating the DSB repair process (van den Boom et al. 2016). USPs play critical roles in ubiquitin-directed signaling by catalytically removing ubiquitin from substrate proteins and multiple USPs. USP7, USP21, and USP48 have been reported to play regulatory roles in HRR (Agathanggelou et al. 2017; Liu et al. 2017; Uckelmann et al. 2018). USP51 modulates NHEJ by reversing RNF168-mediated ubiquitination of histone H2A (Wang et al. 2016). However, the mechanism by which USPs are involved in RIII has not yet been fully elucidated. Our research revealed that USP15 is highly expressed in both HIEC and mouse intestines after irradiation, suggesting its potential role in regulating intestinal DDR. Furthermore, in vitro and in vivo studies have indicated that USP15 inhibitors increase the number of DSBs, the most severe form of DNA damage while promoting apoptosis and clonogenic cell death in intestinal cells after radiation. To date, few articles have reported the direct role that UPS15 plays in the pathogenesis of RIII. One study demonstrated that USP15 could interact with and stabilize a known DNA repair factor called FUS (fused in sarcoma) in hematopoietic stem cells and leukemia cells. Moreover, radiation-induced inflammation triggers redox activation, and cellular redox metabolism is a promising target for mitigating radiation injury. Interestingly, several studies have reported that USP15 can modulate cellular redox reactions in acute myeloid leukemia, Huntington’s disease and diabetic nephropathy (Chan et al. 2023; Niederkorn et al. 2022; Xu et al. 2022). Notably, poly (ADP-ribose) polymerase inhibitors (PARPis) are DNA-damaging agents developed for cancer therapy, and USP15 has been shown to affect the response of cancer cells to PARPis by regulating HRR(Peng et al. 2019). Moreover, UPS4 has been found to promote homologous recombination-mediated repair by regulating the recruitment of the MRE11-RAD50-NBS1 (MRN) complex and CtIP at DNA damage sites, whereas USP11 has been reported to catalyze H2AK119 and H2BK120 deubiquitination and is functionally involved in the DNA repair process upon DNA damage. USP4, USP11, and USP15 are paralogous USPs, as evidenced by their structural organization and sequence similarity (Vlasschaert et al. 2015; Wijnhoven et al. 2015). Collectively, our findings indicate that USP15 may regulate DNA damage repair and RIII by modulating the key regulators of the DDR.

The DDR is a complex signaling system consisting of sensors, transducers, and effectors and is responsible for detecting DNA damage and mobilizing the downstream cascade of DNA reparative mechanisms (Brobbey et al. 2022). ATM acts as an apical sensor and amplifies DNA damage signaling (Lee, et al. 2021). Increasing evidence has demonstrated that PTMs such as phosphorylation, acetylation, methylation, and ubiquitination are critical for ATM activation (Kim et al. 2020; Lee, et al. 2021; Xie et al. 2023). For instance, ATM is immediately autophosphorylated upon DNA damage, triggers a cascade of phosphorylation events on chromatin-flanking DSBs, and recruits downstream effectors, including RAD51 and BRCA1 (Sun et al. 2020). Acetylation of ATM by the Tip60 histone acetyltransferase plays another key role in the activation of ATM upon DNA damage (Sun, et al. 2017). O-linked β-N-acetylglucosamine (O-GlcNAc) is another type of posttranslational modification (PTM) that usually occurs reciprocally with phosphorylation (Miura et al. 2012). Interestingly, ATM is an O-GlcNAc-modified protein, and O-GlcNAcylation affects the ATM-induced DNA damage response (Lee. 2024). Regarding ubiquitination, the MRN protein complex mediate ATM kinase signaling in response to irradiation, and Skp2 has been recently reported to trigger the K63-linked ubiquitination of NBS1 upon DSB and enhances the interaction of NBS1 with ATM, thereby facilitating ATM recruitment to DNA foci for activation (Sheng et al. 2024). Meanwhile, NBS1 and ATMIN directly compete for ATM binding in response to ionizing radiation, and another study has proved that UBR5-mediated ubiquitination of ATMIN is required for ionizing radiation-induced ATM signaling and function(Zhang et al. 2014). However, the direct role of ubiquitination in the activation process of ATM in response to DNA damage remains unknown. In this study, we determined that USP15 and ATM could bind mass spectrometry analysis and Co-IP and GST pull-down assays, respectively. Furthermore, USP15 increased ATM expression by extending its half-life. Specifically, we found that USP15 upregulates ATM by promoting deubiquitination and inhibiting proteasomal degradation. While ATM stability has been previously reported to be affected by ubiquitination, USP15 is, to our knowledge, the first USP to be directly involved in ATM stabilisation.

The domains most frequently represented in USPs include the catalytic core domain, the ubiquitin-like domain (UBL), and the DUSP domain (in USP) (Zhao et al. 2020). The catalytic core domain is a specific region of the enzyme that directly binds to and catalyzes the chemical reaction of the substrate. The UBL domains share the conserved b-grasp fold of ubiquitin; however, they lack the final GG residues and frequently have evolved to different functions. DUSP domains are exclusively found in USPs and may participate in substrate recognition by USPs (Lange et al. 2022). USP15 contains three catalytic core domains (D1, D2, and D3 of the C- terminus), an N-terminal DUSP domain, and two UBL domains, with one UBL domain embedded into the catalytic core domain (Peng et al. 2019). The presence of UBL domains, both inside and outside their catalytic cores, is likely to modulate the enzymatic activity of USP15, recognition of different ubiquitin chain types, and recruitment of ubiquitinated proteins to the proteasome (Das et al. 2021). In this study, by expressing full-length or truncated USP15 mutants, we found that USP15 interacts with ATM through its C-terminal D3 catalytic core domain. The DUSP and UBL domains are typically considered to play a role in mediating USP interactions. However, our results revealed that deletion of the USP15 UBL domain did not affect its binding to ATM. Another study consistently demonstrated that USP15 could interact with BARD1, whereas deletion of its D3 domain (deletion residues 740–981), but not that of the UBL domain, abolished the binding of USP15 with BARD1 (Peng et al. 2019). Therefore, studies on USP15-substrate interaction mechanisms are limited and require further investigation.

Interestingly, phosphorylation status regulates not only the localization of USPs but also their activity. For example, glycogen synthase kinase 3 beta (GSK3β) can directly bind to and phosphorylate USP27X, thereby enhancing the interaction between USP27X and CBX2 (Xing et al. 2023). Moreover, the phosphorylation of USP20 at Ser334 by IRAK1 is required for its ability to induce IL-1β-evoked signaling in vascular smooth muscle cells and vascular inflammation (Zhang et al. 2023). One article reported that the phosphorylation status of USP15 at Thr149 and Thr219 alters its interaction with its partner protein SART3, consequently leading to its nuclear localization and deubiquitinating activity toward the substrate PRP31 (Das et al. 2019). Recently, a growing body of evidence has suggested that USPs are downstream targets of ATM. For example, one study demonstrated that USP24 is stabilised by ATM phosphorylation at S1620 upon DNA damage. Subsequently, increased levels of USP24 lead to p53 stabilisation/upregulation and the subsequent activation of p53 downstream targets (Wang et al. 2021). Another study revealed that USP15 can be phosphorylated by ATM and recruited to sites of DNA damage. Nucleated USP15 then helps retain the key protein BARD1, which is essential for HRR(Peng et al. 2019). Therefore, it will be interesting to determine further whether phosphorylated USP15 enhances the deubiquitination and stability of ATM after radiation exposure, thus potentially forming an ATM/USP15 positive feedback loop in the context of DNA damage.

The above results and discussion clearly indicate that USP15/ATM axis may play an important role in the pathogenesis of RIII. Since target USP15 could activate ATM signalling and then mitigate DNA damage and RIII. Then, the next major and urgent task is to develop USP15-targeted methods as potential countermeasures for RIII.The rapid, dynamic and reversible ubiquitination–deubiquitination process plays a key role in maintaining protein homeostasis, controlling the turnover and biological function of most proteins within the cell, and is thereby critical to almost all biological and pathological processes. Therefore, targeting key elements involved in ubiquitination–deubiquitination could be effective for the treatment of certain diseases. Among them, an emerging and promising approach in regulation of ubiquitination–deubiquitination involves to target DUBs, since suitable regulation of DUBs can necessarily assist in controlling the cell biology and physiology, while DUBs turnover defects can contribute to disease development and progression. For example, since 2015, several DUBs-targeted agents, including VLX1570 (targeting USP14), P22077 (targeting USP7/USP10/USP47), P5091 (targeting USP7/USP47), and spautin-1 (targeting USP10/USP13), have been investigated in the clinic to treat certain types of cancers. Moreover, a variety of DUBs-targeted agents have been tested for their ability to fight against non-cancer diseases, such as neurodegeneration, ataxia, Parkinson’s disease, Huntington’s disease, inflammatory diseases, and even SARS coronavirus infection Notably, MTX652, an USP30-targeted agent, has successfully completed its first Phase I clinical study, and has recently gained clearance from the US FDA to commence a Phase II trial as a potential candidate treatment for mitochondrial dysfunction To date, none USPs-targeted agents has yet been approved for clinical use. However, as another major type of PTMs, discovery of phosphorylation was awarded the Nobel Prize in 1992, and since then, various kinase inhibitors such as Gleevec have been developed and successfully approved as drugs for clinical usage. Therefore, it is reasonable to envision that DUBs, including USP15, represent a hopeful reservoir of therapeutic targets in the future.

Nevertheless, there are still certain challenges as well as limitations that need to be resolved before it can be used for clinical application. First, one major challenge for targeting USP15 for therapeutics is the potential for off-target effects, as the high degree of homology between USP15 and its paralogs USP4 and USP11 will undoubtedly pose a challenge in developing agents that are highly specific for this DUB. Second, although USP15 functions as a tumor-inhibiting factor, its knockout in lung and hepatocellular cancer cells promotes malignant phenotype transformation, including increased proliferation and metastasis (Ren et al. 2023). However, the dual function of USP15 in regulating the progression of different cancers has been determined (Shi et al. 2024). Therefore, before clinical application, other potential molecular regulatory mechanisms of USP15 deserve further investigation. Overall, we believe that USP15 and its target agent have a bright future in the field of RIII treatment and are worthy of further study.

There are still some limitations in this current study. Firstly. the molecular mechanism underlying how radiation could induce upregulation of USP15 is not clear yet. As mentioned above, since increasing number of evidence has suggested that USPs are the downstream targets of ATM, whether an ATM/USP15 positive feedback loop is forming in the context of DNA damage need to be further investigated. Secondly, although our data suggested that USP15 can directly interacts with ATM via its D3 domain, the specific binding sites of USP15 to ATM remain to be further explored, which is also one of the limitations of our study. Moreover, when we designed the experiments of RIII mouse model, we referenced multiple recent studies to ensure our methodology is up to the standard and found out that using a single abdominal exposure to study RIII has been an acceptable common practice. However, animal model with fractionated radiation exposure will undoubtedly better mimic the clinical study, and worthy further study.

## Conclusions

In conclusion, the results of the present study revealed that USP15 functions as a deubiquitinase to regulate DNA damage repair in RIII by opposing the ubiquitination-mediated degradation of ATM. The findings of this study provided novel potential target and new ideas for the treatment of RIII.

## Data Availability

No datasets were generated or analysed during the current study.

## Abbreviations

ATM: Ataxia telangiectasia mutated
BARD1: BRCA1-Associated RING Domain Protein 1
BRCA1: Breast Cancer 1
CHK2: Checkpoint kinase 2
CHX: Cycloheximide
DDR: DNA damage response
DSB: DNA double-strand break
GST: Glutathione S-transferase
HIEC: Human intestinal epithelial cell
HRR: Homologous recombination repai
IB: Immunoblotting
IF: Immunofluorescen
IHC: Immunohistochemistry
IP: Immunoprecipitation
KAP1: Kinesin II associated protein
Lys: Lysine
NHEJ: Nonhomologous end-joining
PARPi: Polymerase inhibitors
PLA: Proximity ligation assay
PTM: post-translational modification
RIII: Radiation-induced intestinal injury
RT: Radiotherapy
UBL: ubiquitin-like domain
USP11: Ubiquitin-specific peptidase 11
USP15: Ubiquitin-specific peptidase 15
USP24: Ubiquitin-specific peptidase 24
USP28: ubiquitin-specific peptidase 28
USP4: Ubiquitin-specific peptidase 4
USP5: Ubiquitin-specific peptidase 5
USP7: Ubiquitin-specific peptidase 7
